# Rationale, design, and methods of electroencephalography-based investigation of the effects of oral desmopressin on improving slow-wave sleep time in nocturnal polyuria patients (the DISTINCT study): protocol for a single-arm, open-label, single-assignment trial

**DOI:** 10.1186/s12894-020-00668-5

**Published:** 2020-07-11

**Authors:** Kazumasa Torimoto, Makito Miyake, Yasushi Nakai, Katsuya Aoki, Nobumichi Tanaka, Kiyohide Fujimoto

**Affiliations:** Department of Urology, Nara Medical University, 840 Shijocho, Kashihara, Nara 634-8522 Japan

**Keywords:** Nocturia, Nocturnal polyuria, Desmopressin, Sleep quality, Slow-wave sleep, Delta power

## Abstract

**Background:**

Nocturia is one of the most bothersome lower urinary tract symptoms and often impairs sleep quality in the elderly. Although previous studies on nocturia have indicated that the successful treatment of nocturia improves sleep quality, most used questionnaires and activity devices to analyze sleep/wake patterns. Therefore, there is little information about the treatment effects of desmopressin on objective sleep quality. The aim of the DISTINCT study is to investigate the change in subjective and objective sleep quality using electroencephalography (EEG) and the Pittsburgh Sleep Quality Index (PSQI) after the administration of desmopressin in patients with nocturia due to nocturnal polyuria.

**Methods:**

A total of 20 male patients, ≥65 years old, with nocturnal polyuria, defined as a nocturnal polyuria index (NPi) (nocturnal urine volume / 24 h urine volume) value ≥0.33, will participate in this study. The participants must have a nocturnal frequency of ≥2 and the first uninterrupted sleep period (FUSP) must occur within < 2.5 h. Desmopressin 50 μg per day will be orally administered before going to bed for 4 weeks. Urinary frequency volume charts (FVC) and EEG will be recorded prior to treatment and at 1 week and 4 weeks after the initiation of treatment. The PSQI will be completed before and 4 weeks after treatment. The primary endpoint is the change from baseline in the mean time of slow-wave sleep (sleep stages N3 and N4) at 4 weeks. The secondary endpoints include the change in the mean value of each sleep variable, the mean delta power during the FUSP, the correlation between nocturnal urinary frequency and slow-wave sleep time, and the change in PSQI score before and after treatment.

**Discussion:**

The DISTINCT study will provide valuable evidence to indicate that oral desmopressin treatment for nocturnal polyuria prolongs the FUSP, resulting in the extension of slow-wave sleep time associated with sleep quality.

**Trial registration:**

The Japan Registry of Clinical Trials (jRCTs051190080). Registered 9 December, 2019.

## Background

Nocturia is a common disease in the elderly. One consequence of nocturia is the complaint of poor sleep quality due to polyuria and nocturnal voids [1]. Nocturia is defined by the International Continence Society as “The number of times urine is passed during the main sleep period. Having woken to pass urine for the first time, each urination must be followed by sleep or the intention to sleep” [2]. The prevalence of nocturnal urinary frequency ≥ 2 is 47% in Japanese residents ≥65 years old [3], 36% in European residents from 60 to 80 years old [4], and 14.2% in the United States (mean age of patients is 46 years) [5]. Nocturia correlates with age, race/ethnicity, medical problems (such as hypertension, diabetes, and stroke), psychological aspects, tasting habits, quality of life, and even mortality [6].

Nocturia causes poor sleep quality by prompting nocturnal voids during the first uninterrupted sleep period (FUSP) [7, 8]. Nocturia increases the number of nocturnal voids, and nocturnal voiding has been shown to be an independent predictor of both self-reported insomnia (75% increased risk) and reduced sleep quality (71% increased risk), along with female gender and other medical psychiatric conditions [7]. Nocturia decreases the FUSP, and the FUSP is a potentially valuable metric that correlates with changes in perceived sleep duration, depth, quality of sleep for the entire night, efficiency, and latency, as evaluated by Pittsburgh Sleep Quality Index (PSQI) [8]. The FUSP has a close relationship with slow-wave, or non-REM, sleep. Sleep stages are divided from 1 to 4, and slow-wave sleep occurs during stages N3 and N4. Stages N3 and N4 commonly occur during the first two sleep cycles (about 180 min after falling asleep) and are considered important for high-quality sleep [9].

We hypothesize that desmopressin treatment for nocturia will improve subjective and objective sleep quality. Desmopressin 25 and 50 μg orally disintegrating tablets are now available worldwide for nocturia and have been shown to decrease the number of nocturnal voids [10]. It is reported that subjective sleep quality, estimated by a quality of life questionnaire, was improved by desmopressin treatment [11]. Desmopressin prolonged the FUSP, suggesting that the slow-wave sleep time might also have been prolonged, although this has not been investigated.

The aim of the DISTINCT study is to use electroencephalography (EEG) and PSQI to investigate the change in subjective and objective sleep quality after the administration of desmopressin to patients with nocturia due to nocturnal polyuria.

## Methods/design

**Fig. 1:**
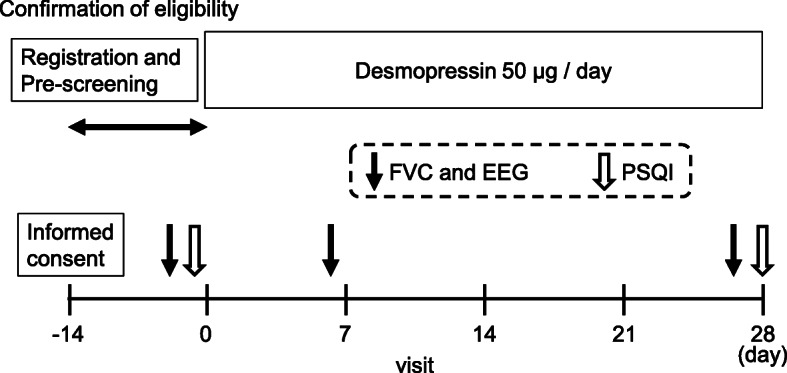
Study design. A total of 20 patients with nocturnal polyuria will participate in this study. After the conformation of eligibility, the enrolled patients will be treated with oral desmopressin 50 μg per day. In the pre-screening period, 2-day frequency volume chart (FVC) and sleep encephalography (EEG) will be recorded and the Pittsburgh Sleep Questionnaire Index (PSQI) will be completed. Before visit Day 0, 2-day FVC and EEG will be recorded. Before visit Day 7 and Day 28, 2-day FVC and EEG will be recorded. On visit Day 28, the PSQI will be completed

This is a single-arm, open-label, single-assignment study. It is an exploratory study to compare subjective and objective sleep qualities after 1 week and 4 weeks of desmopressin treatment for nocturnal polyuria (Fig. 1). Qualifying patients will be males, ≥65 years old, and suffering from insomnia due to nocturnal polyuria, defined as a nocturnal polyuria index (NPi) value (nocturnal urine volume / 24 h urine volume) of ≥0.33 [2].

This study will be conducted at Nara Medical University Hospital in compliance with both the articles of the Declaration of Helsinki (revised in October 2013) and the Ethical Guidelines for Medical and Health Research Involving Human Subjects established by the Ministry of Health, Labor, and Welfare in Japan.

### Planned outcomes

#### Primary endpoint

The primary endpoint is the change in mean time of slow-wave sleep (stages N3 and N4) as evaluated by EEG at 28 days from baseline (Table 1).

**Table 1:** Primary and secondary endpoints of the DISTINCT Study

Primary endpoint	Secondary endpoints
Change in mean time of slow-wave sleep (N3 + N4) as evaluated by EEG at 28 days from baseline	1. Change is PSQI before and after desmopressin administration
	2. Change in nocturnal urinary frequency, the first nocturnal urinary volume, and the FUSP before and after desmopressin administration
	3. Change in sleep time, sleep effiency, and sleep quality as estimated by VAS before and after desmopressin administration
	4. Correlation between slow-wave sleep time (N3 + N4) and PSQI, VAS, FUSP, nocturnal urinay frequency at 28 days from baseline
	5. Change is delta power during the FUSP before and after desmopressin administration

*N1–4* non-rapid eye movement sleep stage 1–4, *EEG* eletroecephalography, *PSQI* Pittsburgh sleep quality index, *FUSP* the first uninterrupted sleep period, *VAS* visual analogue scale

#### Secondary endpoints

The secondary endpoints are listed in Table 1.

##### Safety endpoints

Data on the clinical trial results, vital sign measurements, and adverse events (AEs), especially signs of hyponatremia, will be collected at each visit. The severity, causal relationship to desmopressin, and outcomes of all AEs will be assessed.

### Study population

A total of 20 male patients, ≥65 years old, with nocturnal polyuria, will participate in this study. The participants must have nocturnal frequency ≥ 2 and NPi ≥0.33, which will be recorded by 2-days FVC. Patients will be considered eligible for the study if they fulfill all of the inclusion criteria and none of the exclusion criteria, as defined in Table 2. The investigator will provide a sufficient explanation of the study to each patient and obtain written informed consent prior to the initiation of any study procedures.

**Table 2:** Inclusion and exclusion criteria of the DISTINCT Study

Inclusion criteria	Exclusion criteria
1. Nocturnal urinary frequency is 2 or more.	1. Concentration of serum sodium is less than 135 mEq/L
2. Nocturnal polyuria index is 0.33 or more.	2. Habitual or psychogenic polydipsia
3. FUSP is 2.5 h or less.	3. Heart failure, the history of heart failure, or suspected heart failure
4. Concentration of serum sodium is 135 mEq/L or more.	4. Fluid retention that requires diuretic treatment, whether currently or in the patient’s medical history
5. Creatinine clearance or eGFR is 50 mL/min/1.73m^2^ or more.	5. Antidiuretic hormone incompatible secretion syndrome
6. Patients with BPH or OAB are acceptable if the storage symptoms (urgency and daytime frequency) are well managed and stable for 3 months or more by any treatments or 6 months or more by 5-alpha reductase inhibitors.	6. Moderate or severe renal failure (Creatinine clearance or eGFR < 50 mL/min/1.73m^2^)
	7. Hypersensitivity to desmopressin or the history, whether currently or in the patient’s medical history
	8. Concomitant or scheduled administration of thiazides, thiazide-like diuretics, or loop diuretics
	9. Concomitant or scheduled administration of corticosteroids
	10. Cardiac pacemaker implantation
	11. Judged to be inappropriate by the principal investigator or subinvestigators

*FUSP* the first uninterrupted sleep period, *BPH* benign prostatic hyperplasia, *OAB* overactive bladder, *eGFR* estimate glomerular filtration rate

### Treatment

Patients will be provided with desmopressin 50 μg (as MINIRINMELT® OD Tablets 25 μg / 50 μg; Ferring Pharmaceuticals Co., Ltd., Tokyo, Japan) to be orally administered once daily prior to going to bed. The drug has been approved only for male patients with nocturnal polyuria in Japan.

### Measurements

The schedule of data collection is detailed in Table 3. A window of ±2 days is acceptable.

**Table 3:** Schedule of enrollment, interventions, and assessments

	Study period								
	Pre-screening		Treatment						
**Timepoint:**	Visit 1	Visit 2	Visit 3			Visit 4			Visit 5
	Day −28 to −14	Day −14 to − 1	Day 0	Day 5	Day 6	Day 7	Day 26	Day 27	Day 28
**Enrollment:**									
Eligibility screen	X								
Informed concent	X								
Registration		X							
**Intervention:**									
Desmopressin									
**Assessment:**									
Patient characteristcs	X								
Blood test	X	X				X			X
PSQI		X							X
EEG		X		X	X		X	X	
VAS			X			X			X
FVC		X		X	X		X	X	
Checking for AEs						X			X

*AEs* adverse events

#### Informed consent and registration

The principal investigator or co-investigator will explain the study to each eligible participant, using the informed consent documents. Participants who give written informed consent will be registered in this study. Registration will be closed when the planned total number of participants (20 participants) is reached.

#### Data collection

Patient characteristics will include age, height, weight, and medical history. Blood tests performed at pre-screening will evaluate the following clinical chemistry analytes: serum creatinine, estimated glomerular filtration rate, sodium (Na), potassium, chlorine (Cl), brain natriuretic peptide, fasting blood glucose, white blood cell count, red blood cell count, hemoglobin content, hematocrit, platelet count, total protein, albumin, blood urea nitrogen, aspartate aminotransferase, alanine aminotransferase, alkaline phosphatase, and gamma-glutamyl transferase. Blood tests performed during the treatment period will evaluate Na and Cl.

A mobile EEG device, Sleep Graph® (Proassist, Ltd., Osaka, Japan), will be used for EEG recordings. The device is small, easy to carry, and well-validated by standard methods (polysomnography) [12]. The device records brain-waves during sleep, which indicate sleep stages.

The FVC will be recorded by the patients independently to assess the nocturnal frequency and nocturnal urine volume. The results of the visual analog scale (VAS) will be used as additional indicators of subjective sleep quality on days when FVC and EEG are recorded. The VAS is a measurement instrument that asks patients to rate their sleep from “I could sleep well” (left end of the scale) to “I could not sleep at all” (right end of the scale). Subjective sleep quality will be assessed by the length between the left end and the point at which a patient records their sleep rating.

### Sample size calculation

This study is designed to demonstrate a statistically significant difference in the change from baseline in slow-wave sleep time in patients treated with desmopressin 50 μg with overall power of 80% and an overall Type 1 error rate of 5% (two-sided). The R program was used for sample size calculation. Bliwise et al. reported that in 15 patients studied, 8 patients had one or two voids, and 7 patients had three or four voids, showing a trend for higher amounts of N3 sleep at 81.4 ± 28.8 min vs 48.2 ± 40.7 min, respectively, assuming a total sleep time of 6 h [13]. Based on these data, we assumed 33.2 min as the difference between both groups and 35.7 min as the standard deviation, and determined that data from 13 subjects will be needed to detect a significant difference from baseline. Considering subject withdrawal, missing data, and feasibility, we assume 20 subjects will be sufficient.

### Statistical analysis

#### Primary endpoint

A Wilcoxon matched-pairs signed rank test would be used to compare the mean time of slow-wave sleep (N3 + N4) at Day 26 or 27 with that at baseline.

#### Secondary endpoints

A Wilcoxon matched-pairs signed rank test will be used to compare the mean score of the PSQI at Day 28 with that at baseline, and the mean time of slow-wave sleep (N3 + N4) at Day 5 or 6 with that at baseline. Friedman’s test and Dunn’s multiple comparisons test will be used to compare the change in mean total sleep time, mean sleep efficiency, mean sleep latency, mean VAS, mean time of sleep stages, and delta power during the FUSP from baseline to Day 26 or 27. The dependency between the mean time of slow-wave sleep (N3 + N4), the score of the PSQI, VAS, FUSP, number of voids, delta power during the FUSP, and other baseline characteristics will be analyzed using a linear regression model at Day 28. The significance level for statistical tests will be 0.05 on two sides.

## Discussion

The aim of the DISTINCT study is to investigate the change in subjective (measured by PSQI) and objective (measured by EEG) sleep quality after the administration of desmopressin to patients with nocturia due to nocturnal polyuria. One of the treatment goals for nocturia is to prolong the FUSP because slow-wave sleep, which is important for brain rest, appears early in sleep. Desmopressin, which has world-wide marketing authorization, has been demonstrated as safe and efficacious in prolonging the FUSP. However, the prolongation of slow-wave sleep time following desmopressin use has not been studied. The DISTINCT study addresses this gap in the research.

Additionally, this study will rely on a novel approach for measuring objective sleep quality. The standard method for analyzing sleep stages is polysomnography, which requires placing many sensors on a patient’s head and body, and can be performed only in laboratories. Therefore, it is difficult for nocturia patients who go to the toilet for voiding to undergo polysomnography, which fixes patients to the bed during the sleep period. The portable EEG device used in this study does not restrain patients and was demonstrated as useful in our previous study, which demonstrated the linkage between sleep quality assessed by the PSQI and slow-wave sleep time [14].

This study has several limitations, including a small number of patients, and an open-label, single-arm modality. However, the results will provide valuable evidence by which to demonstrate the positive effects of desmopressin on objective sleep quality, which is associated with the occurrence of deeper sleep stages in patients with nocturnal polyuria.

## Data Availability

Not applicable.

## Abbreviations

FUSP: First uninterrupted sleep period
PSQI: Pittsburgh Sleep Quality Index
EEG: Electroencephalography
NPi: Nocturnal polyuria index
AE: Adverse event
Na: Sodium
Cl: Chlorine
VAS: Visual analog scale
FVC: Frequency volume chart
